# Australian alcohol policy 2001–2013 and implications for public health

**DOI:** 10.1186/1471-2458-14-848

**Published:** 2014-08-15

**Authors:** Steven J Howard, Ross Gordon, Sandra C Jones

**Affiliations:** School of Education, Faculty of Social Sciences, University of Wollongong, Northfields Avenue, Wollongong, NSW 2522 Australia; Department of Marketing and Management, Faculty of Business and Economics, Macquarie University, Sydney, NSW 2019 Australia; Centre for Health and Social Research, Australian Catholic University, 115 Victoria Parade, Fitzroy, VIC 3065 Australia

**Keywords:** Alcohol, Policy, Legislation, Australia, Public health

## Abstract

**Background:**

Despite a complex and multi-faceted alcohol policy environment in Australia, there are few comprehensive reviews of national and state alcohol policies that assess their effectiveness and research support. In mapping the Australian alcohol policy domain and evaluating policy interventions in each of the core policy areas, this article provides a useful resource for researchers. The implications for protecting public health emanating from this mapping and evaluation of alcohol policy are also discussed.

**Methods:**

This review considered data from: published primary research; alcohol legislation, strategies and alcohol-related press releases for all levels and jurisdictions of Australian government; international publications by prominent non-governmental organisations; and relevant grey literature. These were organised and evaluated using the established framework offered by Thomas Babor and colleagues.

**Results:**

Findings indicated great variability in alcohol initiatives across Australia, many of which do not reflect what is currently considered to be evidence-based best practice.

**Conclusions:**

Research showing increasing alcohol-related harms despite steady levels of consumption suggests a need to pursue alcohol policy initiatives that are supported by evidence of harm-reduction. Future initiatives should aim to increase existing alcohol controls in line with suggested best practice in order to protect public health in Australia.

## Background

Alcohol-related health and social harms are well documented [1]. In Australia, recorded annual per capita alcohol consumption stands at 9.89 litres and alcohol accounts for a significant proportion of the total burden of disease and injury (3.3% in 2003) - second only to tobacco in terms as a preventable cause of drug related deaths and hospitalisation [2]. Indeed, alcohol accounts for a considerable number of preventable deaths (over 31,000 between 1992 and 2001) and hospitalisations (over half a million between 1993–94 and 2000–01) [3]. Despite efforts to reduce alcohol-related harms as illustrated in this paper, recent evidence has indicated increasing alcohol-related harms in Australia; while population levels of consumption are relatively stable, there are changing patterns of drinking among sub-groups [4]. Given the burden of alcohol on public health, there is considerable debate on alcohol control policy. However, the depth of research on the effectiveness of some alcohol policies remains limited, especially in the Australian context (although the recently announced International Alcohol Control Study [5] will go some way to increasing understanding). As such, along with the World Health Organization’s [6] identification of ‘best buy’ alcohol control policies for projecting public health (i.e., pricing strategies, limiting availability, controlling marketing), mapping the existing landscape can help inform alcohol policy development and strategy, such as the National Binge Drinking Strategy and components of the Taking Preventative Action national health strategy.

This article contributes to knowledge in three ways. Firstly, it maps the alcohol policy environment in Australia, providing a reference for alcohol researchers. Secondly, it evaluates alcohol policy in Australia according to the Babor et al. [7] framework for effective alcohol policies. Finally, comparing and contrasting the findings with available evidence of best practice, it discusses the implications for future alcohol policy and protecting public health in Australia.

## Methods

This article presents the key findings from a systematic review of alcohol policy in Australia spanning all states, territories, and levels of government. The article provides a synopsis of Australian alcohol policy, and an assessment of strengths and weaknesses in each policy area. The literature review followed the PRISMA protocol for conducting and reporting systematic reviews [8]. Identified literature was then categorised and assessed by utilising the matrix offered by Babor et al. [7], outlining seven broad areas of alcohol policy: 1. pricing and taxation; 2. regulating physical availability; 3. modifying the drinking environment; 4. drink-driving countermeasures; 5. restrictions on marketing; 6. education and persuasion; and 7. treatment and early intervention. Importantly, Babor et al. [7] based their identification of seven key areas of alcohol policy on extensive consultation of the extant research literature, and theoretical assumptions underpinning the seven broad areas – for example, for alcohol pricing and taxation the theoretical assumption is that increasing the economic cost of alcohol relative to alternative products will reduce demand. Therefore, the framework offers a useful, and the most established, tool for identifying and evaluating alcohol policy holistically.

Currently, policy initiatives relating to each area are employed by all national, state, and territory governments in Australia. Local government in Australia usually has a limited, supporting role in relation to some of these (e.g. land use planning controls, enforcement of local laws).

### Inclusion/exclusion criteria

To investigate alcohol policy across these seven areas, literature published from 2001–2013, in English language only, and consisting of published academic primary research and commentaries examining current or previous alcohol policy/strategy in Australia, its effectiveness, and/or correlates of change were included. In addition, grey literature such as published alcohol legislation, strategies and alcohol-related press releases for all levels and jurisdictions of Australian government were included. The review also included grey literature publications by prominent non-governmental organisations; government reports, stakeholder publications, and media reports. It should be acknowledged that there were important developments in Australian alcohol policy introduced prior to the date range for the present review, such as the introduction of random breath testing in 1982, and mid strength beer during the 1990s. This review helps map how alcohol policy has developed during the past decade, identifying areas in which it is robust, and areas in which it may be sub-optimal and providing suggestions for future improvements to protect public health.

### Search strategy

A systematic search for academic literature consisting of primary research or commentaries spanned five electronic databases (see Table 1). Search terms included, but were not limited to, alcohol*, Australia*, polic** legislat*, pric*, tax*, regulat*, avail*, environ*, ban*, minimum, restrict*, density, training, code, enforce*, law*, test*, BAC, licen*, punish*, campaign, intervention, and treatment,^[a]^ and results were limited to records published between 2001 and 2013^[b]^ (see Table 1). From the 1196 results, 187 articles were identified for retrieval by reviewing titles and abstracts, and of these 126 articles were subsequently retained (after removing irrelevant records, and duplicates). Following this, grey literature was identified by searching organisation websites and through Google searches using the same search terms used to search academic databases. This process included identification of alcohol-related legislation, strategies and press releases from national, state, and territorial government websites including all health departments, transportation authorities, liquor and gaming offices, and departments of education in Australia; governmental taskforce reports; key stakeholder publications; and media reports.

**Table 1:** **Electronic database search summary**

Electronic database	Range of disciplines	No. of records returned/retained
Scopus	Chemical sciences, biological sciences, medical and health sciences, physical sciences, psychology, law, economics, human society, education, politics and policy, and more	471/101
Medline	Medicine, nursing, toxicology, nutrition, life sciences, and more	132/39
PsycINFO	Psychology and related disciplines (e.g., medicine, neuroscience, and nursing)	161/42
Web of Science’s Social Science Citation Index	Biological sciences, medical and life sciences, physical and chemical sciences, law, and more	432/109
Web of Science’s Arts and Humanities Citation Index	Sociology, urban studies, communication, criminology, law, nursing, rehabilitation, and more	

*Note:* Web of Science’s Social Science Citation Index and Arts and Humanities Citation Index were searched as one database, thereby generating a single set of records. The final number of articles included in the review after removing duplicates was 147, plus 78 items of grey literature.

This supplemental search of the grey literature identified 78 additional sources. Inter-coder reliability checks were conducted by two researchers on a 20% sample of the literature to check for consistency in inclusion/exclusion, and subsequent categorisation, with disagreements settled by a third reviewer. Once the included literature had been identified it was downloaded into bibliographic software and categorised according to the seven key policy areas identified by Babor et al. [7]. Data extraction was then conducted to enable a summary of Australian alcohol policy in each of seven policy areas to be constructed. The protocol issued by Babor et al. [7] was then used to assess and rate the effectiveness, and research support thereof, of Australian alcohol policy for each of the seven policy areas, with inter-coder reliability checks being conducted during the entire process and disagreements resolved through majority decision (see Table 2). It should be acknowledged that the Babor et al. [7] protocol does have some limitations in that it favours policies for which there is empirical evidence of implementation, and availability of peer reviewed evaluation literature. This means that policy areas for which there is a paucity of evaluation research, or where the design and implementation of policy has been poorly executed, may be ranked lower. However, despite these limitations, this protocol offers the most appropriate framework for reviewing and evaluation alcohol policy in Australia. The following summarises the key findings that emerged^[c]^.

**Table 2:** **Ratings of policy-relevant strategies and interventions**

Strategy or intervention	Effectiveness	Research [**] support	Australian implementation and research notes
**Pricing and Taxation**			
Alcohol Taxes	++	++	Three national taxes (Excise, WET, GST). Research suggests that increasing taxes reduces alcohol consumption and harms – especially so in a strictly volumetric taxation system.
Bans on Price Discounts/Promotions	?	+	Variable by state/territory. Current research evidence is highly contextual
Differential Price by Beverage	+	+	Taxation-based. Research suggests taxes on high-alcohol content beverages can shift consumption to lower-alcohol content options
Special/Additional Taxation	+	+	‘Alcopop’ tax. Research suggests higher alcopop prices can reduce consumption without complete substitution, but unclear impact on harms.
Minimum Price	?	***+***	In development. Limited evidence of effectiveness.
**Regulating physical availability**			
Ban on Sales	+++	+++	Bans limited to particular high-risk areas. Research suggests bans can lead to substantial harm and consumption reductions.
Minimum Legal Purchase Age	+++	+++	18 years of age throughout Australia. Research suggests that enforcement can substantially increase effectiveness.
Hours and Days of Sale Restrictions	++	++	Variable by state/territory. Research suggests effectiveness is tied to whether availability is meaningfully restricted (especially during high-risk times).
Restrictions on Density of Outlets	++	+++	Licensing in all states/territories, but form varies. Research has consistently demonstrated link between outlet density and alcohol-related harms.
Ban on Drinking in Public Places	?	+	Variable by state/territory. Research suggests such bans may displace but not reduce harms.
**Modifying the drinking environment**			
Staff Training in Responsible Service of Alcohol (RSA)	0/+	+++	Required in all states/territories, but who is required to have RSA varies.
Server Liability	++	++	Servers liable in all states/territories, but penalties vary. Research suggests effectiveness is increased with awareness.
Voluntary Codes of Bar Practice	0	+	Voluntary accords agreed locally. Research suggests ineffectiveness when completely voluntary.
Late-Night Lockouts of Licensed Premises	?	+	Highly variable in terms of adoption and form by state/territory. Limited research available.
Enhanced Enforcement of On-Premises Laws	***++***	***++***	Enforcement highly variable. Research suggests effectiveness of most initiatives depend on strength of enforcement, yet research suggests police target individuals more than establishments.
**Drink-driving countermeasures**			
Random Breath Testing	+++	++	Used throughout Australia. Research suggests effectiveness tied to consistency and publicity.
Lowered BAC Limits	+++	+++	<.05 throughout Australia. Research suggests lower BACs lead to higher rates of effectiveness.
Administrative Licence Suspension	++	++	Immediate suspension occurs throughout Australia, but threshold for suspension varies by state/territory. Research suggests effectiveness is tied to immediacy and consistency.
Low Blood Alcohol Content for Young Drivers	+++	++	Zero tolerance for learner or provisional drivers – additional measures vary by state/territory. Research suggests especially effective for those below legal drinking age.
Graduate Licensing for Novice Drivers	++	++	Occurs throughout Australia and permits lower BAC for young drivers.
Designated Drivers and Ride Services	0	+	Voluntary and variable across states/territories. Research suggest may encourage higher rates of consumption by passengers and has no impact on rates of alcohol-related road accidents.
Severity of Punishment	0/+	++	Punishment for drink driving variable by state/territory. Limited evidence of effectiveness in reducing alcohol-related road accidents.
**Restrictions on marketing**			
Legal Restrictions on Exposure	+	+++	Alcohol advertisements can only be shown in M, MA and AV classification periods. Research suggests dose–response effect for young drinkers, but small effect on per-capita levels of consumption.
Alcohol Industry Voluntary Self-Regulation Codes	0	++	Self-regulatory code implemented, funded, and administered by alcohol industry. Research repeatedly suggests ineffectiveness and repeated evidence of code breaches.
**Education and persuasion**			
Classroom Education	0	+++	Drug and alcohol education incorporated into all state/territory curricula, in variable forms. Research suggests little long-term effects on drinking behaviours.
Mass Media Campaigns	0	+++	Extensive resources committed to ongoing mass media campaigns. Research suggests little evidence of impact on consumption levels.
Warning labels/signs	0	***+***	Imminent on voluntary basis, ongoing discussion around whether to legally require. Research suggests labels create little change in drinking behaviours.
**Treatment and early intervention**			
Brief Intervention with At-Risk Drinkers	+	+++	Sporadic use and often tied to workplace programmes. Research suggests that these measures can be effective.
Mutual Help/Self-Help Attendance	++	++	Various mutual-help programmes (e.g., AA) throughout Australia.
Mandatory Treatment of Repeat Drink-Drivers	+	++	Currently limited to trialling of alcohol interlocks in some states/territories. Research suggests time-limited effects.
Medical/Social Detoxification	+	++	Sobering-up centres used in Australia, which focus on short-term harm reduction. Research suggests short-term benefits but limited effect on long-term consumption unless combined with other treatment options.

**Research support is based on Australian research studies or studies including Australia.

*Note:* Adapted from Babor et al. (2003, 2010) for the Australian context. Effectiveness refers to evidence for reducing alcohol consumption and/or alcohol-related problems (0 indicates a lack of effectiveness; 0/+indicates mixed evidence that suggests effectiveness depends upon strength of enforcement; + indicates evidence for limited effectiveness; ++ indicates evidence for moderate effectiveness; +++ indicates evidence for a high degree of effectiveness; ? indicates that no known controlled studies have been undertaken or insufficient evidence exists). Research support refers to the quantity and consistency of available evidence (0 indicates that no effectiveness studies have been undertaken; + indicates one or two well-designed effectiveness studies have been undertaken; ++ indicates several effectiveness studies have been undertaken, but no comprehensive reviews were available; +++ indicates sufficient studies conducted and a comprehensive review or meta-analysis was available). Bolded initiatives indicate those that are not yet instituted in Australia but are imminent or subject to ongoing discussion.

## Results

### Pricing and taxation

Pricing is one of the most effective alcohol harm minimisation policy levers available, with a strong evidence base suggesting that increases in the price of alcohol reduce consumption, and associated harms. Although the price of alcohol in Australia is relatively high compared to other developed countries, this may be due to market conditions, high living costs, and the strong Australian dollar, rather than due to policy effects. A number of factors affect the cost of alcohol, the most prominent of which is taxation. Australia’s three national taxes on alcohol account for a large share of the estimated $6 billion the Australian Government receives as a result of alcohol-related production and consumption [9–11]. A volume-based Excise Tax (Excise Tariff Act 1921) that increases according to the strength of alcohol is applied to all beer, pre-mixed alcoholic beverages and spirits. A mix of deliberate concessions and some unintended loopholes have served to create numerous exceptions to the volumetric nature of this tax. Spirits, ready-to-drink (RTD) products, and flavoured cider are subject to excise based the volume of alcohol in the products. Notably, some spirits (mainly Brandy) are subject to a concessional rate (effectively creating differential pricing by beverage). The excise rate for beer is lower than that on spirits and depends upon its packaging (e.g., draught or bottle). Wine, and traditional cider, in contrast is subject to a value-based Wine Equalisation Tax (WET; A New Tax System [Wine Equalisation Tax] Act 1999). Under the WET, wine is taxed at a flat rate of 29% of its wholesale value [11]. After the Excise or WET rate has been applied, a further value-based GST (A New Tax System [Goods and Services Tax] Act 1998) is then applied to all alcoholic beverages at a flat rate of 10% [10]. As these taxes are regulated and collected by the Commonwealth Government, alcohol taxation is one of the few areas of national uniformity in Australian alcohol policy.

Recent research has questioned the merits of this complex and inconsistent system of alcohol taxation, suggesting instead that significant health gains and cost savings could be achieved with a strictly volumetric taxation system, whereby all alcohol products would be taxed according to their alcohol content [12–14]. While public discussions on alcohol taxation reform have increased in recent years, these have not yet resulted in tangible policy change. Instead, the Australian government has favoured the adoption of special taxes to influence problematic patterns of alcohol consumption. These additional taxes have commonly been levied against alcoholic beverages identified as disproportionately consumed by youths or at-risk groups. A recent example is the passage of the Excise Tariff Amendment (2009 Measures No. 1) Bill, which addressed a loophole that meant that spirit-based, pre-mixed, ready-to-drink beverages (commonly termed ‘alcopops’), were taxed differently to spirits. The Bill targeted alcopops specifically due to their identification as a drink of choice for Australian youths, particularly young Australian women [9–11]. In support of these measures, research has suggested their ability to influence preferences and purchasing behaviours [15], to increase taxation revenue, and to reduce pure alcohol consumption [16].

In 2009, a national taskforce on preventative health recommended that the Australian government make further improvements to the alcohol taxation system, and also explore the feasibility of setting a minimum price on alcohol. Public consultations for the development of a national minimum price per standard drink of alcohol were then undertaken in 2011 [17] consistent with Australia’s National Alcohol Strategy 2006–2011 [18], which highlights the Australian Government’s continued commitment to investigating price-related levers aimed at reducing harmful drinking practices. It is true that alcohol taxation is higher in Australia than many other developed nations (such as the UK) for beer and spirits (but not wine), and there is taxation of specific products such as alcopops in place. However, further policy measures such as the introduction of minimum unit pricing, hold the potential to further protect public health.

Although the taxation of alcohol occurs on a national level in Australia, individual states and territories influence alcohol pricing through their regulation of discounts and promotions. In most cases, these regulatory powers have specifically targeted promotional activities that promote unsafe or irresponsible consumption of alcohol, although this pattern of consumption is not clearly defined and its encouragement is almost impossible to prove. Most states have legislated their right to prohibit promotional activities involving alcohol at reduced prices, although the specificities and types of measures to achieve these aims vary. For instance, New South Wales (NSW) specifically states that generally a discount promotion of over 50% should be undertaken with caution and with risks being properly assessed on whether it will encourage immoderate consumption of alcohol [19]. Queensland prohibits discounted price promotions in licensed premises that may encourage immoderate consumption. Less specific stipulations prohibiting alcohol promotions from encouraging immoderate drinking are in place in Victoria, and Western Australia. In the Northern Territory the Liquor Act includes a clause stating that it may be necessary to prohibit or limit promotional activities but provides no further clarification. In contrast, Tasmania and the Australian Capital Territory have no current legislation that explicitly prohibits promotional activities involving alcohol sold at reduced prices.

Overall, our assessment is that Australian policy in this area is stronger than other developed countries like the UK [7], but there are inconsistencies in taxation and the level of control over price reductions and promotions is weak and difficult to enforce. Moving forward, consistent taxation policies and measures such as minimum unit pricing of alcohol can further strengthen the policy environment.

### Regulating physical availability

Beyond taxation, regulation of advertising, and delivery of education and persuasion interventions, alcohol policy is largely legislated by state and territory governments – a diffusion of control that has rendered Australian alcohol policy varied and in a constant state of change. Contributing to this variability are differences in alcohol consumption, alcohol-related harms and the political context that ultimately motivates legislative change across individual states and territories [10]. To illustrate this diversity, several Australian states and territories have recently rewritten their alcohol legislation in the context of increasing rates of alcohol-related harm (i.e., ACT Liquor Act 2010, NSW Liquor Act 2007, NT Liquor Act 2010). Nevertheless, all Australian states and territories have altered their alcohol legislation to some extent in the past decade, with revisions commonly aiming to regulate the physical availability of alcohol as a means to reduce alcohol-related harms. However, other recent revisions have aimed to reduce the regulatory burden on business (see the red tape reduction bill in Queensland discussed later) that could potentially threaten public health oriented provisions in alcohol policy.

Licensing is the most commonly used mechanism for regulating the availability of alcohol. Licensing regulates who is able to sell alcohol and places conditions on where (the density of outlets), when (trading hours) and how (license conditions) alcohol can be sold – all of which have been shown to correlate with harmful consumption practices [13, 20–26]. All Australian states and territories currently have a licensing system in place, although they vary in the categories of licenses available (e.g., Victoria has 12 license types to Northern Territory’s five) and the conditions attached to each type of license. Although there is some consistency in licence conditions across Australia, such as the minimum legal purchase age (18 years) and mandatory responsible service of alcohol training, which from 2014 will be administered at the Commonwealth level [27], a number of fundamental differences exist. For instance, states and territories differ in their position on the consumption of alcohol by minors. Most states ban the consumption of alcohol by minors in licensed or public premises. However, Tasmania and South Australia (SA) permit minors to drink in non-dry public areas under the supervision of a responsible legal guardian. Northern Territory (NT) extends this by permitting minors to consume alcohol in licensed premises with proper guardian supervision.

Another point of divergence is the normal trading hours attached as a condition to licenses. States and territories vary in: whether they have revised trading hours for Sundays (Queensland and Tasmania do not); the earliest time that establishments are permitted to serve alcohol (i.e., 5 am for NSW, Tasmania, SA; 6 am for Western Australia; 7 am for Victoria; 10 am for Queensland, NT); and the latest time an establishment is permitted to serve alcohol on a standard license (i.e., midnight for NSW, SA, Queensland, Western Australia, Tasmania; 11 pm for Victoria; 10 pm for NT). In addition, while most states and territories offer extended (e.g., NSW, SA, Victoria, Western Australia, Tasmania), or even 24-hour (e.g., SA) alcohol trading licenses, other states have taken a different route to alter their late-night trading environment. For example, in 2009 the Queensland Government placed a moratorium on granting licenses that extend the trade of alcohol beyond midnight or before 5 am [28]. However, a recent move in June 2013 to reduce red tape associated with obtaining a liquor licence in Queensland has seemingly relaxed the regulatory environment [27]. Similar measures in NSW mandate that all extended trading licensees maintain a minimum of six hours continuous closure each day (NSW Liquor Legislation Amendment Act 2008). Similarly, SA recently proposed mandatory closure of all alcohol-licensed establishments between the hours of 4 am and 7 am (SA Liquor Licensing (Miscellaneous) Amendment Bill 2011), although this was ultimately defeated in favour of harsher penalties for alcohol-related transgressions [29]. States also place considerable focus on encouraging economic development, with South Australia and New South Wales recently introducing simplified small venue liquor licenses to encourage a small venue culture in Adelaide [30, 31]. This focus on urban development may not always be congruous with protecting public health or controlling alcohol related social problems.

Another common means for regulating the availability of alcohol is limiting the locations in which alcohol can legally be consumed. In particular, Australian states and territories continue to differ in their treatment of alcohol consumption in public. While all states and territories have established some form of restricted area alcohol-free or dry zones (typically in areas such as public roads, parks and beaches), the extent of these provisions varies. In 2008, NT placed a wholesale ban on alcohol in particular high-risk areas (e.g., the town of Katherine) [32]. In contrast, other states (Victoria and Tasmania) have given authority to the local councils to impose these restrictions. Despite these various initiatives, research has suggested only moderate levels of compliance, perhaps related to a need to redirect current enforcement strategies from individual drinkers to the supplying establishment [33].

The topic of social/secondary supply of alcohol to minors in private premises has generated significant debate, and policy changes, in recent times. For example, supply of alcohol to minors on private premises by persons other than the minor’s adult guardians is prohibited in the Northern Territory since 2011, New South Wales since 2007, Queensland and Tasmania since 2009, and Victoria since 2011 [34]. In the Northern Territory, Queensland and Tasmania the legislation also proscribes that supply must occur responsibly and under supervision [35]. Yet in South Australia, the Australian Capital Territory, and Western Australia secondary supply of alcohol to minors in private premises is unregulated. Furthermore, there are some issues with these laws worth considering. It is not illegal to supply alcohol to your own children, or to other’s children provided parental or guardian permission is granted, which means that the policy often does not prevent social supply of alcohol to minors. In addition, such laws are very difficult to police, as supply of alcohol to minors often takes places in private homes in which enforcement agencies are not present.

There is strong research support for controlling alcohol availability to reduce alcohol consumption and related harms, suggesting that this is an important area of focus in Australia. A number of changes in recent years in the area of regulating the physical availability of alcohol appear to have somewhat strengthened policy in this area, especially when compared with countries like the UK and much of the EU. However, our assessment is that policy in this area is inconsistent. The number of alcohol licenses, and the duration of opening hours remain problems in urban areas, and enforcement of policies such as those pertaining to social supply of alcohol to minors is problematic. An emerging challenge is the growth in packaged (i.e. take-away) liquor sales, which now represent around 80 per cent of all alcohol consumed, because the bulk of this is consumed in the home or other unlicensed premises where there are relatively few controls on servers and drinkers. Overall, while there have been policy developments that have regulated alcohol availability to an extent, inconsistencies between states and territories suggest that further improvements could be made.

### Modifying the drinking environment

Another function of licensing the sale of alcohol is to exercise control over the drinking environment, typically accomplished by placing conditions on alcohol licenses. Nearly all Australian states and territories require responsible service of alcohol (RSA) training for staff involved in the service of alcohol as a condition of alcohol licensure. Some states extend this condition further, requiring this training for all staff and security (i.e., NSW, Victoria, Tasmania, Australian Capital Territory). In contrast, in SA only one ‘approved responsible person’ with RSA training is required to be on duty (SA Liquor Licensing Act 1997). Western Australia (WA) has legislated the ability to flexibly apply this training requirement as needed (WA Liquor Control Act 1988). From 2014, nationally administered and regulated RSA training will supersede state competencies for this area of policy [27].

Another common feature of alcohol legislation is the recent inclusion of server liability. In all states and territories it is now illegal for licensees and alcohol service staff to sell or supply alcohol to an intoxicated person [9]. Infractions are subject to a fine, carrying a maximum penalty usually in the thousands of dollars for staff and tens of thousands for licensees. In addition, most states have extended this liability to other patrons of the establishment who supply alcohol to an intoxicated person (usually incurring a fine approximately half that of staff), with NT being a notable exception.

An additional measure aimed at modifying the drinking environment is the adoption of late night lockouts. These lockouts aim to restrict the movement of patrons between establishments by setting a time (prior to closing) after which entry or re-entry is no longer permitted [9], yet the use of lockouts remains highly variable. For instance, Queensland has a state-wide 3 am lockout for all late-trading premises [36], whereas other states (NSW, SA) have various locally agreed and/or voluntary lockouts. Further, Victoria trialled and abandoned legislated 2 am lockouts in Melbourne in 2008, whereas WA has recently begun trialling them [37]. Although many states have supported their lockout plans with evidence of harm reduction [38, 39], there is somewhat limited formal evidence of their effectiveness due to a lack of comprehensive evaluation studies, and given that lockout polices are often one component of a range of programmes aimed at curbing late night alcohol related problems [9]. Indeed, a recent study identified that although restrictions in opening hours were associated with a sustained lower assault rate in Newcastle CBD, there was no evidence that lockouts in isolation of reductions in opening hours were effective in nearby Hamilton [40].

A number of additional powers are also currently in place to facilitate the enforcement of laws in licensed premises (e.g., barring orders, emergency closure of licensed premises, banned drinker registers, increased penalties for infractions). The success of these measures, however, is reliant upon their effective enforcement. One unique enforcement measure has been NT’s creation of a banned drinker register in 2011, such that all individuals purchasing alcohol must have their ID scanned at liquor outlets [41]. Drinkers who had been banned for violations of the NT Liquor Act 2010 were refused service by alcohol service staff. However, the NT government recently scrapped the banned drinker registers, and introduced a draconian mandatory treatment system instead. In Victoria, new powers introduced in 2012 permit police officers, protective services, and gambling and liquor inspectors to seize and tip out alcohol from persons they reasonably believe are under the age of 18 years [42], and similar legislation is in place in NSW. Powers of seizure of alcohol under circumstances that contravene the Liquor Control Act apply in Western Australia and Queensland; and power of seizure of things by investigators apply in the Northern Territory, and the Australian Capital Territory, although these do not specifically stipulate that they apply to the seizure and tipping out of alcohol in possession of minors.

Another measure is NSW’s ‘three strikes’ legislation, which states that after three violations (from a prescribed list of offences) license conditions can be imposed, a license can be suspended or cancelled and/or a moratorium on a new license can be invoked (NSW Liquor Amendment (3 Strikes) Bill 2011). NSW also runs the ‘Alcohol Linking Program’, which is an intervention that transmits research into practice to enhance police enforcement of liquor laws through the use of data-based feedback to police and licensees about alcohol related crime following drinking on specific licensed premises. Through a series of standard questions asked by police of people involved in an alcohol fuelled incident, the programme effectively ‘links’ incidents where the offender, victim or driver may have consumed too much alcohol in a licensed premises before that incident, and then uses this information to map links where offenders and victims consumed alcohol, and to inform changes in serving practices and environmental design in licensed premises to potentially reduce alcohol related harm [43]. Despite these measures, research suggests application, or enforcement can be inconsistent, often targeting individual problem drinkers rather than the violating establishment [33].

Current Australian alcohol policy in relation to modifying the drinking environment is reasonably well developed with national mandatory responsible server training (often not comprehensively present in other countries), and reasonably strong powers for police and licensing agencies. However, there is some evidence that enforcement is not consistent, and the research evidence for the effectiveness of strategies such as lockouts is mixed. Therefore, current policy in this area is only moderately robust and effective.

### Drink driving countermeasures

Given the serious risks associated with impaired driving, laws prohibiting driving under the influence of alcohol are entrenched within Australian alcohol policy. Despite states and territories each regulating their roads and driver licensing, there is much consistency with respect to drink-driving legislation. For instance, all Australian states and territories require drivers to have their blood alcohol content (BAC) below .05. They also mandate that learner and provisional drivers have no alcohol in their system while operating a vehicle. All states and territories also enforce suspensions for first and subsequent drink-driving infractions, employ an alcohol interlock scheme for serial offenders (which requires a breathalyser test in order to start the car) [44], and utilise random breath testing to enforce these regulations.

Despite these similarities, there are also a number of points of divergence related to drink-driving legislation. One notable example is the BAC threshold leading to immediate license suspension. Immediate license suspension occurs on a first offence for a BAC of .08 in NSW, SA, NT and WA (reduced in 2011), .10 in Queensland (reduced from .15 in 2011), .07 in Victoria and .05 in Tasmania and Australian Capital Territory (ACT). There is also a large degree of variability in the specific penalties for drink-driving infractions across Australia. While increasing monetary penalties for drink driving offences has been a common mechanism to reduce alcohol-related road accidents, evidence suggests that this may in fact be ineffective [45]. For example, research indicates that after doubling the drink driving penalties in NSW in 1998 the rate of single-vehicle accidents increased and the aggregate level of road accidents remained largely unchanged. As a result, Australian governments have increasingly sought viable alternatives to reduce rates of drink driving. One notable example is Queensland’s adoption of cumulative disqualification periods for repeat drink drivers in 2008, which requires that in the case of multiple charges (e.g., drink-driving, then driving while disqualified) the first charge must be carried out in full before the second charge begins [46]. This is in contrast to the typical system where multiple charges are served concurrently. Regardless of the specific measures adopted, the extensive government resources committed to combatting drink driving (e.g., multi-million dollar advertising campaigns) suggests that this will continue to be an area of priority for Australian governments.

The research evidence on the efficacy of drink driving counter measures is relatively strong, and current Australian policy is robust in this area. For instance, Australia has a lower BAC level, and tougher restrictions on drink driving, and resultant penalties than other nations such as the UK. As such, our assessment is that alcohol policy in this area in Australia is strong.

### Restrictions on marketing

Research has repeatedly shown the impact that alcohol marketing can have on preference and purchasing behaviours [15, 25, 47]. Despite this impact, there are currently no bans on alcohol advertising in Australia, although a number of laws and codes at the national level regulate its content and exposure (Trade Practices Act 1974; Commercial Television Industry Code of Practice 2010; Commercial Radio Code of Practice 2004; Outdoor Media Association’s Code of Ethics 2011), in addition to state and territory fair trade legislation [9]. These laws and codes regulate when alcohol advertisements can be shown and contain requirements for honest and ethical advertising. For instance, the Commercial Television Industry Code of Practice 2010 dictates that alcohol advertisements may only be shown in M, MA, and AV classification periods; although they are also permitted during broadcasts of sporting events that occur on weekends and public holidays.

Nevertheless, Australia’s system for regulating alcohol advertising can be best described as quasi-regulatory given that the centrepiece of this regulatory system is the Alcohol Beverages Advertising Code (ABAC), which is implemented, funded, and administered by the alcohol industry [9, 48]. The stated aim of this code is to ensure that alcohol advertising presents responsible drinking [49], although there is growing evidence of code violations [48, 50].

Features of this code include: not targeting children and adolescents; not encouraging excessive consumption of alcohol; not implying that alcohol consumption will lead to a significant change in mood; not depicting an association between alcohol consumption and vehicle operation; and not depicting the consumption of alcohol as contributing to various forms of success [49]. In addition to regulating television and radio advertisements, the ABAC scheme also pertains to Internet advertisements, retail advertisements, promotion of alcohol at events (in conjunction with state regulations), and the packaging of alcoholic beverages (in conjunction with national regulations). Although controlling alcohol marketing remains a point of focus for regulators, this has centred on guidelines and voluntary regulation rather than legislation. For instance, liquor promotion guidelines were introduced in NSW in 2013, with principles pertaining to not appealing to minors, using indecent or offensive promotions, encouraging immoderate drinking, using emotive language to encourage consumption, offering extreme price discounts, and conducting promotions not in the public interest such as associating alcohol with discrimination, crime or violence [19]. However, these new guidelines largely mirror existing voluntary guidelines and have been criticised for their scope (i.e., they predominantly apply to licensed venues and, like price-discounting restriction, are not applied to packaged alcohol outlets) and lack of enforcement (i.e., rely on breaches being observed by authorities, and subsequently charged and prosecuted) ^[d]^.

Furthermore, more recent discussions have centred on health promotion activities such as warnings labels on alcohol packaging albeit the alcohol industry successfully lobbied for a delay on their introduction [51], rather than on regulatory measures on alcohol promotion activities. Australia has a long history of industry working to pre-empt government restrictions on alcohol marketing by introducing voluntary measures – it has been argued that these measures serve to portray the industry as responsible while avoiding more effective policy interventions [52]. For example, governmental reviews of alcohol advertising regulation have typically been followed by industry ‘modifications’ to the self-regulatory code, which have subsequently been shown to be ineffective [53]. Whilst there is some level of control in current Australian policy with regards level of exposure to alcohol marketing, the current codes are largely self-regulatory and strongly influenced by industry. Given the self-regulatory orientation of policy restricting alcohol marketing in Australia, and the research evidence suggesting that the regulatory system is ineffective, our assessment is that alcohol policy in this area is weak.

### Education and persuasion

With respect to education and persuasion interventions on alcohol in Australia, it is important to acknowledge that such measures are massively dwarfed by pro-drinking messages from alcohol marketing. There has been significant public discourse regarding the inclusion of warning labels on alcohol packaging in Australia over the past five years, but so far only limited policy change. In 2011, Australia’s largest brewers, wine makers and spirits s opted to voluntarily place warning labels on their alcohol products [54]. These labels feature a pictogram indicating that pregnant women should not consume alcohol, as well as one of a number of interchangeable statements such as ‘it is safest not to drink while pregnant’, ‘kids and alcohol don’t mix’, or ‘is your drinking harming yourself or others?’ Given that these measures have been criticised as ambiguous and ineffective by some experts [55, 56], the Commonwealth Government is deliberating over the need for legislation mandating the presence and form of these warnings, as well as the inclusion of a nutritional information panel that list energy levels in kilojoules [56].

Australian alcohol education strategies have also commonly featured large-scale mass media campaigns to emphasise the negative aspects of risky drinking behaviours. Common topics have included binge drinking, anti-violence, alcohol related harms, drink-driving and underage drinking. For instance, the Australian Government’s National Binge Drinking Strategy [57] committed $20 million to highlight the consequences of excessive alcohol consumption. However, these campaigns have generally been found to be effective at raising awareness but ineffective at changing behaviour. Furthermore, notably absent from campaigns has been a focus on raising awareness of the National Health and Medical Research Council drinking guidelines. For example, the evaluation of the ‘Drinking Choices’ National Alcohol Campaign, which targeted teenagers aged 12–17 years and their parents, found high levels of awareness, few attitudinal effects, and no change in teen drinking behaviours (other than an increase in binge drinking among females). A focus on binge drinking, is also evident at state level. For instance, SA’s ‘Drink too much, you’re asking for trouble’ campaign, and WA’s ‘Alcohol. Think Again’ campaign focuses on alcohol related harm. Furthermore, a number of states have focused their campaign efforts on alcohol-related violence, such as: Queensland’s ‘Know your limits’ campaign, which included the use of YouTube clips aimed at raising awareness of the link between alcohol and violence; and Tasmania’s ‘Getting through the night without a fight’ campaign which involved the use of Facebook and a mobile app (Mate Minder) that provides the ability to track friends, request for friends to ‘come find me’ and let you know if friends arrived home safely.

While these campaigns primarily target the reduction of the most prevalent risky behaviours in current drinkers, Australian governments have also aimed at prevention in future drinkers through classroom education. In 2004 the Australian Government released the second edition of its Principles for School Drug Education framework [58], outlining best-practice principles for incorporating drug education into the school curriculum. Following this lead, all state and territory education departments have incorporated some form of drug and alcohol education into their curriculum. For instance, in NSW drug education is taught in every government primary and secondary school from Kindergarten until Year 10 [59]. The SA Department of Education and Community Services has provided staff with drug and alcohol education units to use in Year 5, 6, and 7 classrooms [60]. The WA Department of Education and Training has outlined its School Drug Education programme [61], which includes a dedicated website with curriculum materials and resources for teachers, parents and students. The programme also involves the parents of students, and local communities, therefore taking a more strategic and community development approach to alcohol education.

Despite the variability in the exact requirements and specifications of the drug and alcohol education programme, it is clear that these prevention efforts remain central to governments’ harm-reduction strategies. There is a reasonably high focus on alcohol education and persuasion campaigns in Australia, despite the limited evidence that these have a significant impact on reducing alcohol consumption and related harms. This may be changing given that NSW recently closed the Alcohol and Other Drugs branch in the Department of Education and purchased an industry-developed curriculum. This influence of industry on alcohol education, in addition to the extant research evidence questioning the efficacy of such intervention strategies, leads us to evaluate Australian policy in this area to be weak. However, no formal evaluation of alcohol education and persuasion initiatives in Australia has yet been conducted. There is a reasonably strong focus on alcohol education and persuasion as a pillar of Australian alcohol policy, but the evidence base for its effectiveness is weak, leading us to rank this as a prominent but not entirely evidence-based component of the current policy environment.

### Treatment and early intervention

An additional means for reducing the negative effects of harmful drinking in alcohol dependent drinkers, and preventing at risk drinkers from experiencing further harm, is through the provision of various treatment, routine screening and brief intervention options. However, it has been suggested that this is an area in which Australia has not performed particularly well given the various screening, early identification and interventions that were trialled, yet ultimately abandoned, in the 1980s and 1990s [9]. Nevertheless, one area where these interventions have been consistently applied is in the workplace. Workplace programmes commonly provide high-risk drinkers with an opportunity to access treatment options that have research-based evidence of effectiveness. However, the exact nature of these measures often varies from company to company, consistent only in the minimum practice standards laid out by operational health and safety legislation.

Research on the impact of brief interventions by primary care providers suggests substantial health gains and cost savings [13, 62]; however, legislators have appeared to minimise support for specific treatment or rehabilitation efforts. A prime example is the proliferation of sobering up centres across Australia [9], which are used to temporarily hold publicly intoxicated individuals that are being disorderly. Of particular note is the short-term harm-reduction role this confers on enforcement officials, rather than providing intoxicated individuals with an opportunity for intervention [9]. However, in the Northern Territory, mandatory alcohol treatment has been introduced through which people who are taken into police custody for intoxication three or more times in two months are referred to a compulsory programme of assessment and treatment. Strategies in the programme include treatment in secure residential facilities, community management including income management, life skills and work readiness training [63]. This high involvement programme may be a reflection of the prevalent alcohol problems in the Northern Territory. However, the policy has been criticised as being discriminatory against Aboriginal people, and ignoring issues with problem drinkers who never enter the judicial system [64]. An updated programme for involuntary treatment for alcohol dependence was also introduced in NSW in 2012 [65]. Although the presence of sporadic alcohol-related treatment initiatives suggests that legislators recognise the need for treatment and early intervention for problem drinkers, it is unclear whether this area will become an increased priority in the future.

The research support for alcohol treatment and early intervention, particularly brief interventions, is reasonably strong. However, formal national programmes of brief interventions have only recently been introduced in a limited number of countries. Therefore, it may be some time before evaluation research is available to comprehensively assess the effectiveness of this approach. Whilst efforts have been made in Australia to strengthen policy in this area, provision of services can be sporadic, and there are inconsistencies in intervention approaches. Therefore, our assessment is that policy in this area in some areas is moderately strong (for example workplace interventions), but could improve quite considerably through comprehensive resourcing, and consistent design, delivery and evaluation of interventions.

## Discussion

This paper maps the landscape of Australian alcohol policy between 2001 and 2013, illustrating the breadth of policies and initiatives across seven key policy areas [7]. Examination of this landscape suggests that the Australian policy environment is complex, with Commonwealth, state, and territory governments having competence over different policy areas. This has created considerable variation in policy throughout Australia. Although there is a requirement under a federal system to have a degree of flexibility in alcohol policy development, the current landscape does not optimise public health interests (see Table 2). There is ample evidence to guide governments in developing and implementing alcohol policies that will be effective in reducing public health harms. Yet, despite this, successive governments have been unwilling to introduce these evidence-based policies. There may be a number of reasons for this including opposition to robust policies from the alcohol industry; a pro-drinking culture in Australian communities – facilitated by ubiquitous alcohol marketing, affordable pricing and easy access and availability; and a lack of national coordination, accountability, and strategic governance in relation to alcohol policy, particularly since the Ministerial Council for Drug Strategy was scrapped in 2011.

It is encouraging that some states such as South Australia have now incorporated a public health provision in their alcohol legislation [66], but this is sporadic, and policy implementation that protects public health is often inconsistent. As an example, although many Australian states and territories have attempted to regulate the availability of alcohol for minors, alcohol consumption by minors is subject to fewer restrictions in Tasmania, SA and NT (despite research suggesting that even adult-supervised alcohol consumption by minors results in higher levels of alcohol-related harms than zero tolerance policies [67]. Furthermore, there is little evaluation of the efficacy of alcohol control policy in Australia, with limited research in some policy areas (e.g., marketing, education) suggesting current policy is ineffective.

## Conclusions

This underscores the need for research on the impact and population responses to alcohol control policy in Australia. Relating to WHO’s ‘best buy’ policies of pricing, regulating availability, and marketing control [6], it is clear that Australia currently has moderate controls over alcohol pricing, some limitations on alcohol availability (although these are being weakened, as in the case of alcohol being sold in Victoria’s supermarkets) [68], and limited controls over alcohol marketing. Therefore, policymakers and regulators’ attention should focus on strengthening policy in accordance the evidence base, and with the WHO best buy recommendations in order to protect public health in Australia. Indeed, recent research canvassing opinions of alcohol experts in Australia identified pricing policies such as volumetric taxation and minimum unit pricing, and regulation of alcohol advertising as top national priorities [69]. This may require incorporating public health provisions as a core pillar of alcohol policy, similar to countries like Scotland [70], to facilitate more effective outcomes. Furthermore, public support, which is currently lacking for some measures such as minimum unit pricing for alcohol [71], will also need to be gained for more robust and effective alcohol policy in Australia to emerge.

## Endnotes

^a^A full list of search terms is available from the authors upon request.

^b^This article is based upon an earlier literature review from 2001–2011 (a ten year search span common in systematic review research). The date span in this article was expanded up to 2013 to enable the inclusion of contemporary research, and policy changes.

^c^A full list of references identified in the literature search is available by the authors on request.

^d^Liquor Promotion Guidelines apply to ALL licensed premises BUT state “A distinction can be made between promotions offering alcohol to be consumed immediately on a licensed premises and promotions offering alcohol that which may be stored for consumption later away from the premises. As a result, the extent to which each principle in this document applies to different licence types will vary accordingly”.
